# Construction of a shuttle expression vector for lactic acid bacteria

**DOI:** 10.1186/s43141-019-0013-4

**Published:** 2019-11-18

**Authors:** Tejinder Kaur, Praveen P. Balgir, Baljinder Kaur

**Affiliations:** Department of Biotechnology, Punjabi University, Patiala, 147002 India

**Keywords:** Lactic acid bacteria, Plasmid vector, Expression vector, Heterologous gene expression, Green fluorescent protein, Bile salt hydrolase

## Abstract

**Background:**

Lactic acid bacteria (LAB) are a diverse group of Gram-positive bacteria, which are widely distributed in various diverse natural habitats. These are used in a variety of industrial food fermentations and carry numerous traits with utmost relevance to the food industry. Genetic engineering has emerged as an effective means to improve and enhance the potential of commercially important bacterial strains. However, the biosafety of recombinant systems is an important concern during the implementation of such technologies on an industrial scale. In order to overcome this issue, cloning and expression systems have been developed preferably from fully characterized and annotated LAB plasmids encoding genes with known functions.

**Results:**

The developed shuttle vector pPBT-GFP contains two theta-type replicons with a copy number of 4.4 and 2.8 in *Pediococcus acidilactici* MTCC 5101 and *Lactobacillus brevis* MTCC 1750, respectively. Antimicrobial “pediocin” produced by *P*. *acidilactici* MTCC 5101 and green fluorescent protein (GFP) of *Aequorea victoria* were successfully expressed as selectable markers. Heterologous bile salt hydrolase (BSH) from *Lactobacillus fermentum* NCDO 394 has been efficiently expressed in the host strains showing high specific activity of 126.12 ± 10.62 in *P. acidilactici* MTCC 5101 and 95.43 ± 4.26 in the case of *L. brevis* MTCC 1750, towards glycine-conjugated bile salts preferably as compared to taurine-conjugated salts.

**Conclusion:**

The present article details the development of a LAB/LAB shuttle expression vector pPBT-GFP, capable of replication in LAB hosts, *P. acidilactici* MTCC 5101, and *L. brevis* MTCC 1750. Pediocin and GFP have been used as selectable markers with the efficient production of heterologous extracellular bile salt hydrolase. Thus, the constructed vector pPBT-GFP, with its ability to replicate in multiple hosts, low copy number, and stability in host cells, may serve as an ideal tool for improving LAB strains of commercial value using genetic engineering.

## Background

Lactic acid bacteria (LAB) are historically defined as a heterogeneous group of Gram-positive, microaerophilic, non-sporulating, and low G + C microorganisms that ferment hexose sugars to primarily produce lactic acid. This functional classification includes a variety of industrially important genera, including *Enterococcus*, *Lactobacillus*, *Lactococcus*, *Leuconostoc*, *Oenococcus*, *Pediococcus*, and *Streptococcus* species [1]. These bacteria are commonly found in diverse natural habitats and are used in commercial production of a variety of fermented food products, macromolecules, enzymes, and metabolites [2–4]. The seemingly simplistic metabolism of LAB has been exploited throughout history, for value addition and preservation of foods and beverages in nearly all societies, dating back to the origins of agriculture [5]. Nowadays, LAB have gained significant importance as probiotics, which when consumed aid in the regulation of microflora present in the human gut. In order to widen industrial applications of LAB, an effective way is to improve strain characteristics using genetic engineering techniques. Worldwide research is focused on finding ways to enhance heterologous expression of foreign proteins in the LAB. Plasmids present in LABs provide the opportunity to derive vectors which can be used for various purposes like gene knockouts, amplification, substitution, insertion, multiplication, and expression of heterologous proteins.

For further industrial level applications of such processes, various strategies like the use of high-copy number plasmid vectors, strong constitutive promoter, regulated strong promoters, and extracellular secretion along with scale up are being developed [6]. The genetically enhanced LABs need to be checked for their stability and propagation as well as to their ability to deliver the desired products. Economic viability of such living modified organisms (LMOs) along with their safe generally recognized as safe (GRAS) status are considerations that determine their industrial applications.

Biosafety considerations dictate the choice of safe selectable markers to play an important role in food-grade vector systems. Thus, the use of antibiotic resistance genes as markers are not an option and need to be strictly avoided [7]. Most of LAB secrete β-galactosidase and frequently generate revertants upon the use of an antibiotic resistance gene as a selectable marker. Due to this, there is difficulty in screening out the transformants on media containing X-gal and antibiotics and attempts to find more appropriate ways of screening transformants are continuing [8–10].

In the case of Gram-positive bacteria, the introduction of shuttle vector systems has greatly facilitated gene structure-function studies. However, the construction and range of application of such vectors available to date for Gram-positive bacteria are very inadequate in flexibility and variety. The major problems encountered include the inability to survive in new host strains, high molecular weight leading to low copy number, limited availability of restriction sites for the cloning of novel fragments, instability resulting from large size of fragment inserts, limited host interchangeability, etc. [11, 12]. Thus, recent research in this field has focused on designing of novel, versatile, and efficient shuttle vector systems for Gram-positive bacteria including lactic acid bacterial strains.

Pediococci are comprised of a diverse group of Gram-positive homofermentative LAB, found as saprophytes on vegetable material. These bacteria have been used in the fermentation of vegetables and meats at industrial level [13, 14]. *Pediococcus acidilactici* MTCC 5101 is a GRAS LAB strain capable of secreting pediocin CP2. Biosafety assessment of this strain revealed it to have a non-hemolytic, catalase-negative, gelatinase-negative, and DNase-negative phenotype (unpublished data) and thus can serve as an attractive host for producing recombinant proteins in a biohazard free system. This strain harbors a 9.2 kb plasmid pCP289 which provides an opportunity to design a vector based on its molecular genetic properties. Nucleotide sequence analysis of pCP289 (accession no. KY886451) indicated the presence of a set of genes for pediocin production and transport, which is consistent with the sequence data of pediocin producing pediococcal strains previously reported in literature [13, 15, 16]. The operon consists of the gene *pedA* for the production of prepediocin, a precursor which matures to pediocin CP2 by the removal of a N-terminal peptide, gene *pedB* encodes bacteriocin immunity protein, *pedC* gene product is known to facilitate transport along with PedD, which is the major protein involved in the translocation of pediocin as evident from its homology to various transport proteins. The leader peptide of PedA protein is processed by the respective ABC transporter Ped D protein whose proteolytic activity has been found to be associated with the first few amino acids in cytoplasmic domain. PedC protein consists of a short N-terminal part located in the cytoplasm and a large domain downstream of the transmembrane sequence, located extracellularly. PedC and PedD proteins have been known to play common roles in the maturation and extracellular secretion of pediocins [17–19]. The presence of secretory signal and transport proteins makes the plasmid an attractive resource for vector development with application for the production of therapeutics, food additives, or any immunogenic peptide [16, 20].

Many LAB species can be efficiently transformed by electroporation and have thus become amenable to generate recombinants [12]. Very few plasmids of genus *Pediococcus* have been studied in detail so far, and there is a limited availability of GRAS grade antibiotic resistance free, expression vectors for pediococcal strains [21–24]. Thus, it was proposed to develop a vector keeping in mind the biosafety aspect for industrial applications involving the use of *Pediococcus* and other LAB strains. The present work is aimed at the development of a refined expression system for genetic engineering of pediococci, equipped with biosafe recombinant selection markers and different replication origins to broaden the spectrum of transformable LAB species.

## Methods

### Bacterial strains, plasmids, and culture media

Bacterial strains and plasmids used in this work are listed in Table 1. *P. acidilactici* MTCC 5101 and *L. brevis* MTCC 1750 strains were routinely grown on MRS medium under microaerophilic conditions at 37 °C without shaking. *E. coli* DH5α and K12 were cultured using Luria–Bertani broth medium with vigorous shaking at 37 °C. For agar plates, 1.5–2.0% agar was added to the respective medium. The antibiotic concentrations used were 300 mg/ml erythromycin and 100 mg/ml ampicillin for *E. coli* and 5 mg/ml erythromycin for LAB. Growth media and antibiotics were purchased from HiMedia Laboratories. DNA polymerase, restriction, and ligation enzymes were purchased from Bangalore Genei and New England Biolabs.

**Table 1:** Bacterial strains and plasmids used in the study

Strain	Plasmid	Relevant characteristics	Origin or reference
*Pediococcus acidilactici* MTCC5101	pCP289	Ped^+^, isolated from chilly pickle	Kaur and Balgir [16]
*Escherichia coli* DH5α	pLES003	Shuttle Vector, Amp^R^, Erm^R^	Wada et al. [24]
*Escherichia coli* K12	pMK-RQ	dam^+^, dcm^+^, tonA, rec^−^, Kan^R^	GeneArt, Germany
*Lactobacillus brevis* MTCC1750	–	Isolated during Sinki production	MTCC, Chandigarh

### DNA and PCR manipulation

Nucleic acid manipulations and general cloning procedures were performed according to Sambrook et al. [25]. Plasmid DNA purification was carried out with a High-Speed Plasmid Mini Kit (Geneaid, Taiwan). Plasmid pCP289 from *P. acidilactici* MTCC 5101 was sequenced via next-generation sequencing on Illumina MiSeq platform (Xcelris Labs Ltd., Ahmedabad, India). PCR amplification was as described by Yan et al. [26] and performed with Thermal Cycler (Techne, UK). Table 2 lists the details of PCR-amplified DNA fragments. Restriction digestion and ligation reactions were carried out as per manufacturer conditions. PCR and restriction products were detected by electrophoresis on 1.5% (w/v) agarose gel containing ethidium bromide and photographed by Gel Doc system (Biorad, USA).

**Table 2:** List of PCR primer pairs used for amplification

Primer pair, forward/reverse	Primer sequence (5′-3′)^a^	Template DNA	Target gene	Amplicon length (bp)
pedF pedR	BamHI-GGATCCTATCTAACTAATACTTGACA SphI-GCATGCCGCGAGGATTTCAC	Plasmid pCP289	*pedA-D*	3541
oriF oriR	SacI-GAGCTCATGGTAGCCTCTG KpnI-GGTACCGCTTACATTAACTT	Plasmid pCP289	*repB,ori*	1557
gfpF gfpR	KpnI-GGTACCAGTGTGTTGATAGTGCAGTAT BamHI-GGATCCTTATTTATATAATTCATCCATAC	Plasmid pMK-RQ	*gfp*	867

^a^Restriction enzyme sites are underlined

### Construction of recombinant plasmid and strains

**Fig. 1:**
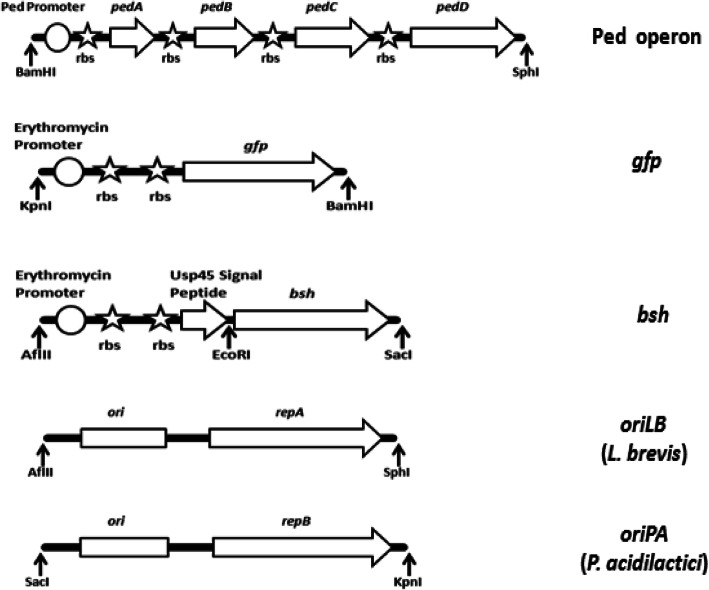
DNA fragments designed for vector construction

DNA fragments designed for vector construction are depicted in Fig. 1. Plasmid pCP289 was used as a template for PCR amplification of pediocin operon and replication origin (*oriPA*) fragment. Pediocin operon was digested with BamHI and SphI and ligated to *oriLB* fragment extracted from plasmid pLES003 after double digestion with SphI and AflII generating fragment I. *oriPA* and *oriLB* consists of RepB and RepA replication proteins and “ori” iteron regions. *oriPA* fragment was double digested with SacI and KpnI and then ligated to synthetic *bsh* fragment with SacI and AflII restriction sites resulting in fragment II. Gene fragments encoding BSH and GFP were artificially synthesized (GeneArt, Germany) as per codon choice of *P. acidilactici* and received as separate inserts in vector pMK-RQ. The nucleotide sequence for *bsh* gene of *L. fermentum* NCDO 394 was retrieved from NCBI vide accession no. JQ293998 and was optimized as per the codon usage of *P. acidilactici* followed by synthesis. Fragment II was constructed by ligation of *bsh* gene sequence to another synthetic sequence (GeneArt, Germany) comprised of constitutive erythromycin (*ery*) gene promoter and *L. lactis* Usp45 signal peptide after restriction digestion with *AflII* and *EcoRI* enzymes. Fragment III comprised of coding gene sequence for GFP, which was synthesized from GeneArt as per codon choice of *P. acidilactici* and received as an insert in vector pMK-RQ. It was retrieved after double digestion with restriction enzymes KpnI and BamHI. Fragments I and II were ligated together after digestion with AflII, and fragment III was then incorporated to generate the shuttle vector pPBT-GFP. This plasmid was electroporated into the *P. acidilactici* MTCC 5101 and *L. brevis* MTCC 1750 using BTX600 Electro Cell Manipulator [27]. The resulting strains showing the Ped^+^, BSH^+^, and GFP^+^ phenotype were screened on MRS agar plates.

Further, the nucleotide sequence of the constructed vector was subjected to restriction analysis using Sequence Manipulation Suite or SMS (http://www.bioinformatics.org/sms2/). The unique sites for restriction enzymes in pediocin operon and *gfp* marker genes were analyzed to be used as insertion points for cloning heterologous genes.

### Determination of plasmid copy number and segregational stability

Plasmid copy number refers to the number of copies of a plasmid present per chromosome in a bacterium. The plasmid copy number was determined by the Avogadro number method described (https://cels.uri.edu/gsc/cndna.html). Plasmid segregational stability was determined as the fraction of culture that maintained the test plasmid after growth for 100 generations without any selective pressure [28, 29]. Cultures were assessed for plasmid maintenance at 0, 50, and 100 generations, respectively, by analyzing 100 randomly selected colonies for the plasmid-borne phenotype.

### Assessment of selectable markers and heterologous genes

#### Bacteriocin activity assay for pediocin

Well diffusion assay was performed to assess the bacteriocin activity in transformed strains [30, 31]. MRS hard agar (1% w/v) was overlaid with MRS soft agar (0.75% w/v) seeded with approximately one million cells of indicator *Enterococcus faecalis*. Thirty microliters of heat-inactivated culture supernatant of transformed *P. acidilactici* MTCC 5101 and *L. brevis* MTCC 1750 was added to wells on agar plates and incubated at 37 °C for 24 h. After overnight incubation, the antimicrobial activity was demonstrated by clear zone around the wells. The diameter of the inhibition zone was measured after the incubation of strains (24–72 h) in appropriate conditions.

#### Green fluorescent protein

The expression of GFP was detected by the spread plate method and epifluorescence microscopy [32]. Overnight grown cultures of transformed *P. acidilactici* MTCC 5101 and *L. brevis* MTCC 1750 were diluted and plated on MRSA plates and incubated at 37 °C for 18–24 h. The plates were then observed under UV light for fluorescence and photographed. For epifluorescence microscopy, dilution of overnight-grown bacterial suspensions was smeared on microscopic slides and production was examined with a fluorescent microscope equipped with a modular filter cube with excitation and emission filters for fluorescence detection and a photographic unit (Leica, Germany).

#### Bile salt hydrolase (BSH) activity

BSH activity was measured by the direct plate method described by Dashkevicz and Feighner [33] with a few modifications and ninhydrin biochemical assay [34]. For plate method, MRS agar plates containing 0.5% bile salt mixture were streaked with overnight grown cultures of *P. acidilactici* MTCC 5101 and *L. brevis* MTCC 1750 with incubation at 37 °C under anaerobic conditions for 48–72 h. BSH activity was indicated by the presence of hydrolyzed products of the salts, viz. cholic acid or deoxycholic acid that precipitated in the agar medium in and around bacterial colonies [35, 36].

The ninhydrin biochemical method described previously by Liong and Shah [34] was used with a few modifications to determine specific BSH activity. It is based on determining the amount of amino acids released from conjugated bile salts [36, 37]. Since BSH in this study was secreted into the media, cell disruption steps were omitted and 0.1 ml of cell-free supernatant was mixed with 0.8 ml 0.1 M sodium phosphate buffer (pH 6.0) and 0.1 ml of 50 mM/l of respective conjugated bile salt (GC, TC, and TDC) and incubated at 37 °C for 30 min. This was followed by an immediate addition of 0.75 ml of 15% (w/v) trichloroacetic acid to 0.75 ml aliquots of mixture and centrifugation at 13,000*g* at4 °C for 10 min. To 1 ml of the supernatant, 2 ml ninhydrin reagent [0.5 ml 1% ninhydrin in 0.5 M citrate buffer (pH 5.5), 1.2 ml 30% glycerol, 0.2 ml 0.5 M citrate buffer pH 5.5) was added. The mixture was kept in a boiling water bath for 30 min and subsequently cooled in ice water. Lastly, the absorption was recorded at 570 nm and the amount of product formed was estimated from a calibration curve generated using glycine or taurine separately. One unit of BSH activity was defined as the amount of enzyme that liberated 1 μM amino acid from the substrate per minute. The specific activity was defined as the number of units of activity per milligram of protein. The Lowry method was used to measure the protein concentrations in the supernatant using bovine serum albumin (BSA) as standard [38]. All experiments in this study were performed in triplicate. BSH activity of each sample was measured against glycocholate (GC), taurocholate (TC), and taurodeoxycholate (TDC) substrates [36].

### Nucleotide sequence accession numbers

The plasmid pCP289 nucleotide sequence was deposited in the GenBank database vide accession number KY886451. For the sequence of pPBT-GFP, see Additional file 1.

## Results

### Construction of expression vector pPBT-GFP

**Fig. 2:**
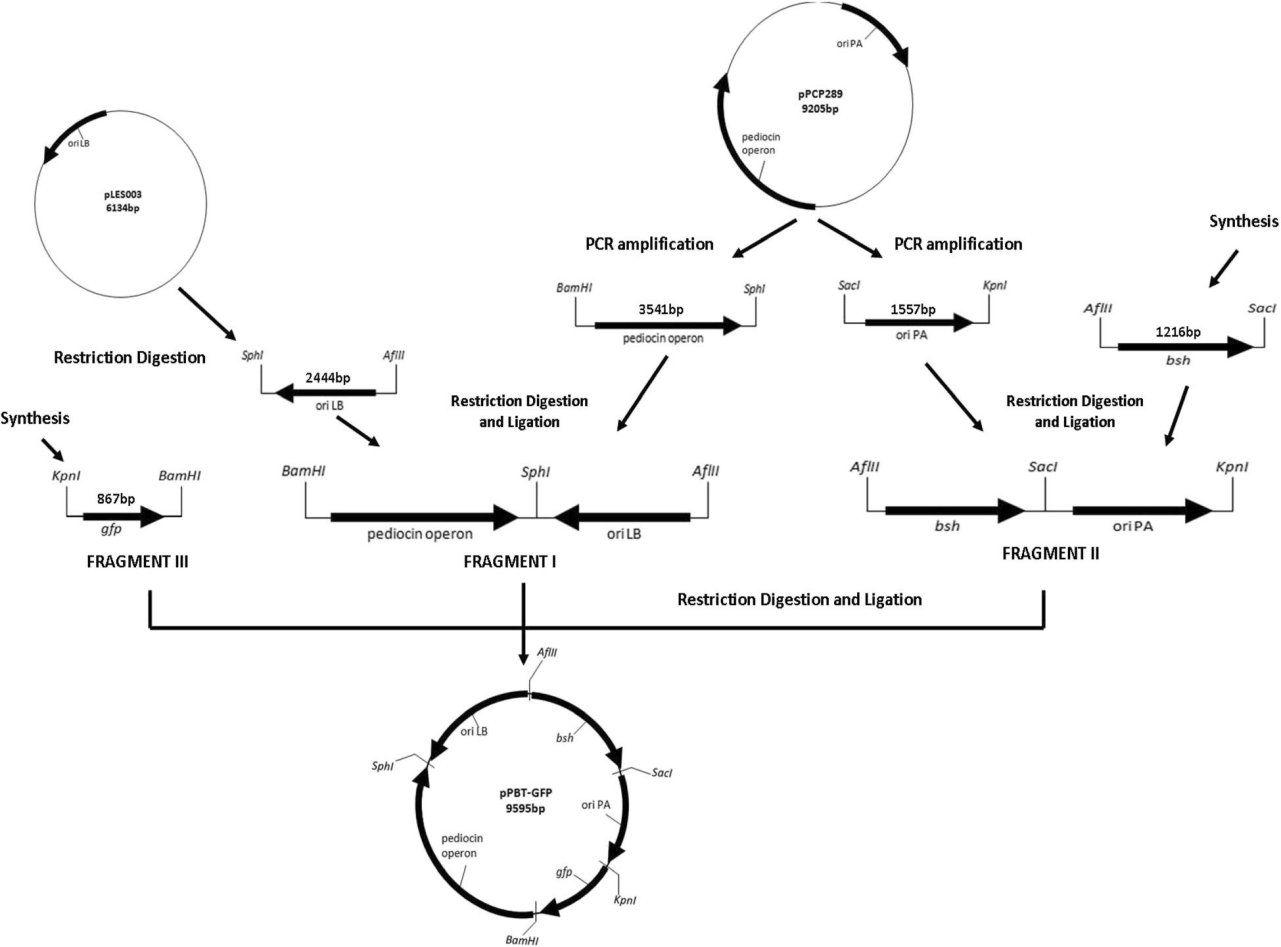
Schematic representation of the construction of pPBT-GFP vector

Schematic representation of construction protocol followed by a LAB shuttle vector pPBT-GFP is shown in Fig. 2. Template plasmids, pCP289 (*P. acidilactici* MTCC 5101), pLES003 (*L. brevis* MTCC 1750), and pMK-RQ (*E. coli* DH5α) were extracted from respective strains and analyzed by agarose gel electrophoresis (Additional file 1: Figure S1). Similarly, PCR amplified and restriction digested fragments, namely pediocin operon, *oriPA*, *oriLB*, *bsh*, and *gfp* were also analyzed on gel (Additional file 1: Figure S2). “Fragment I” was generated by ligation of 3.5 kb pediocin operon from pCP289 and 2.4 kb *oriLB* from pLES003. Similarly, 1.2 kb *bsh* was ligated to 1.5 kb *oriPA* resulting in “fragment II”. Fragments I and II were ligated together followed by the incorporation of “fragment III” containing 0.8 kb coding sequence for *gfp* gene. The size of the resulting vector is 9.6 kb. It contains the dual replication origins from plasmids pCP289 and pLES003 and is able to replicate efficiently in *P. acidilactici* MTCC5101 and *L. brevis* MTCC1750 host strains. Genes for production and expression of pediocin and green fluorescent protein act as recombinant selection markers. The expression of heterologous bile salt hydrolase gene from *L. fermentum* NCDO394 was successfully achieved in *P. acidilactici* MTCC 5101 and *L.brevis* MTCC 1750.

**Fig. 3:**
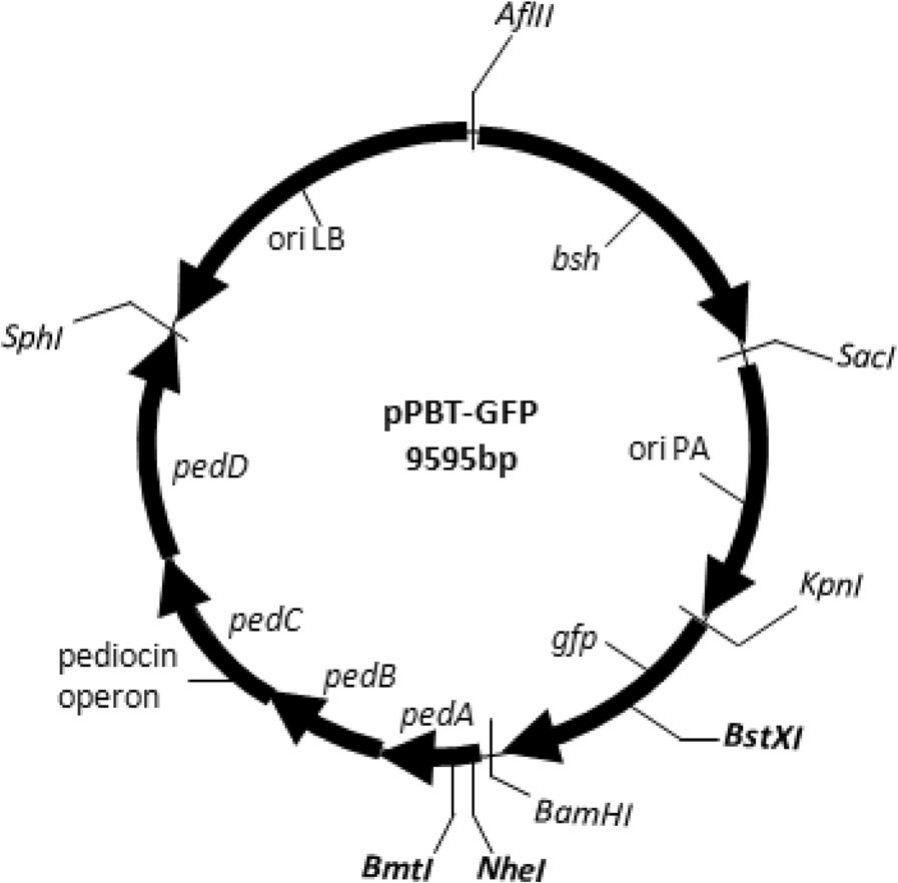
Schematic model of shuttle vector pPBT-GFP showing potential insertion sites for cloning of heterologous inserts. Unique restriction enzyme sites for insertion by suppression of selectable markers are in bold case

For further cloning heterologous inserts in vector pPBT-GFP, insertion sites located within the selectable marker genes are an effective means to select transformants. Insertion will lead to the disruption of marker gene function and related phenotype in transformed cells, which can be used to screen out the recombinant cells. Therefore, restriction analysis of pPBT-GFP vector was carried out using SMS which indicated the presence of unique enzyme sites within pediocin gene cluster. The insertion sites in any of the pediocin producing genes *pedA* or *pedD* that is essential for translocation of pediocin can be efficiently used to suppress Ped^+^ phenotype. NheI and BmtI sites within gene *pedA* and XbaI site in *pedD* can be used for insertional cloning. A unique site for BstXI is present in *gfp* gene for insertional activation of this selectable marker. Moreover, all the DNA fragment inserts in the shuttle vector pPBT-GFP have been cloned using cohesive-end restriction enzyme sites and can be easily replaced with desirable heterologous inserts with similar enzyme sites (Fig. 3).

### Plasmid copy number and segregational stability

Shuttle vector pPBT-GFP showed a PCN value of ~ 4 copies per cell in *P. acidilactici* MTCC 5101 and ~ 3 copies per cell in *L. brevis* MTCC 1750. Although transformation efficiency was found to be comparable to the reported studies involving electro-transfomation in LAB strains [36, 39], plasmid pPBT-GFP has a low copy number and displayed a segregational stability of up to 50 generations in both the host cells (Table 3).

**Table 3:** Transformation efficiency, copy number, and segregational stability of pPBT-GFP

Strain	Transformation efficiency (cfu/ug plasmid DNA)	Copy number	Segregational stability (No. of generations)
*P. acidilactici* MTCC 5101	2.9 ± 0.1X10^4^	4.4	50
*L. brevis* MTCC 1750	1.6 ± 0.1X10^5^	2.8	50

### Bacteriocin activity

**Fig. 4:**
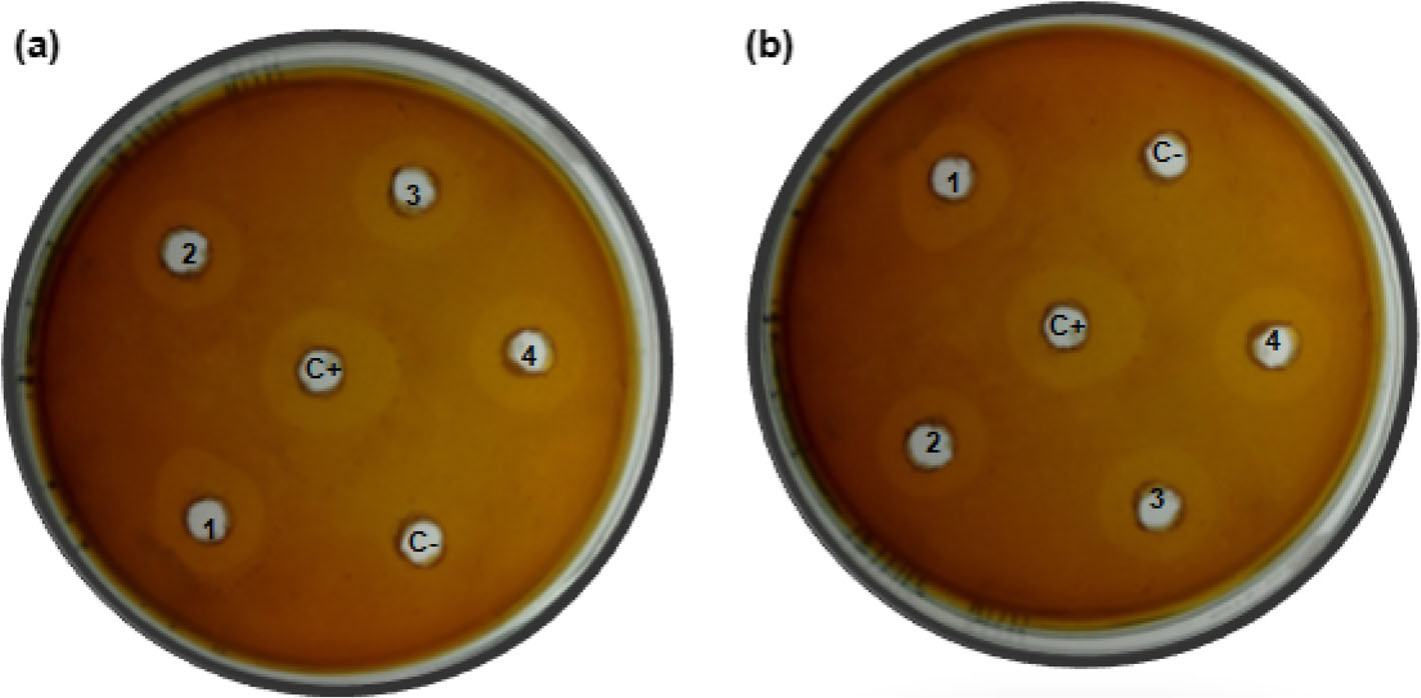
Bacteriocin agar well diffusion assay showing the zone of inhibition against the indicator *E. faecalis*. **a** 1–4: *P. acidilactici* MTCC 5101 transformed with pPBT-GFP. **b** 1–4: *L. brevis* MTCC 1750 transformed with pPBT-GFP. *C+* control native *P. acidilactici* MTCC 5101, *C−* plasmid cured *P. acidilactici* MTCC 5101

To check for pediocin CP2 production in transformed *P. acidilactici* MTCC 5101 and *L. brevis* MTCC 1750, standard agar well diffusion assay was carried out using *E. faecalis* as the indicator strain. Non-transformed *P. acidilactici* MTCC 5101 was used as a control. A definite zone of inhibition was observed in the plates after 24 h. Figure 4 shows the zone of inhibition against the indicator strain and Table 4 lists the diameter of the zone. The transformed and control bacterial strains showed similar inhibition zones depicting efficient expression, production, and secretion of genes of pediocin operon.

**Table 4:** Zone of inhibition of pediocin against *E. faecium*

Strain	Zone of inhibition
	Diameter^a^ (mm)
Plasmid cured hosts (negative control)	0
*P. acidilactici* MTCC 5101 (positive control)	18 ± 0.5
*P. acidilactici* MTCC 5101 with pPBT-GFP	17 ± 0.7
*L. brevis* MTCC 1750 with pPBT-GFP	21 ± 0.5

^a^Values are mean of triplicate ± standard error of means

### Expression of green fluorescent protein

**Fig. 5:**
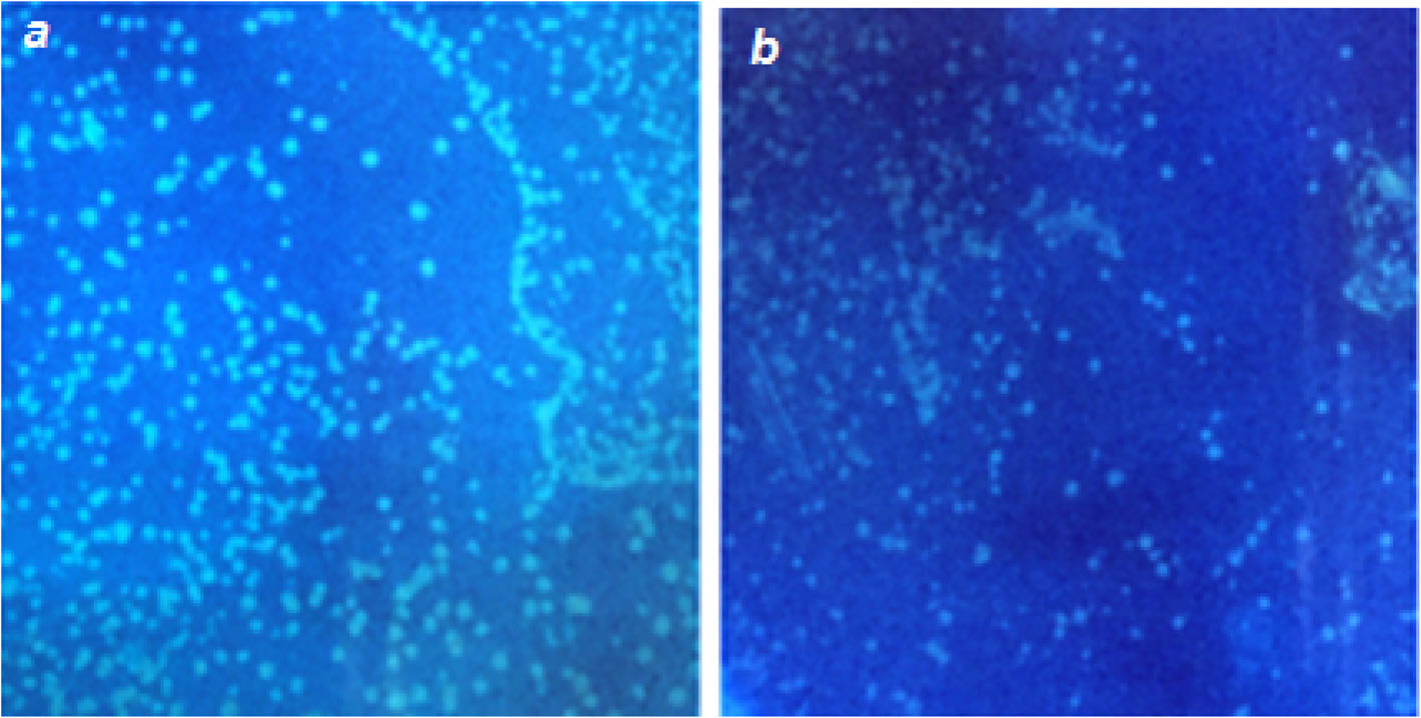
Detection of fluorescence in bacterial colonies on MRSA plates under UV light. **a** *P. acidilactici* MTCC 5101. **b** *L. brevis* MTCC 1750

**Fig. 6:**
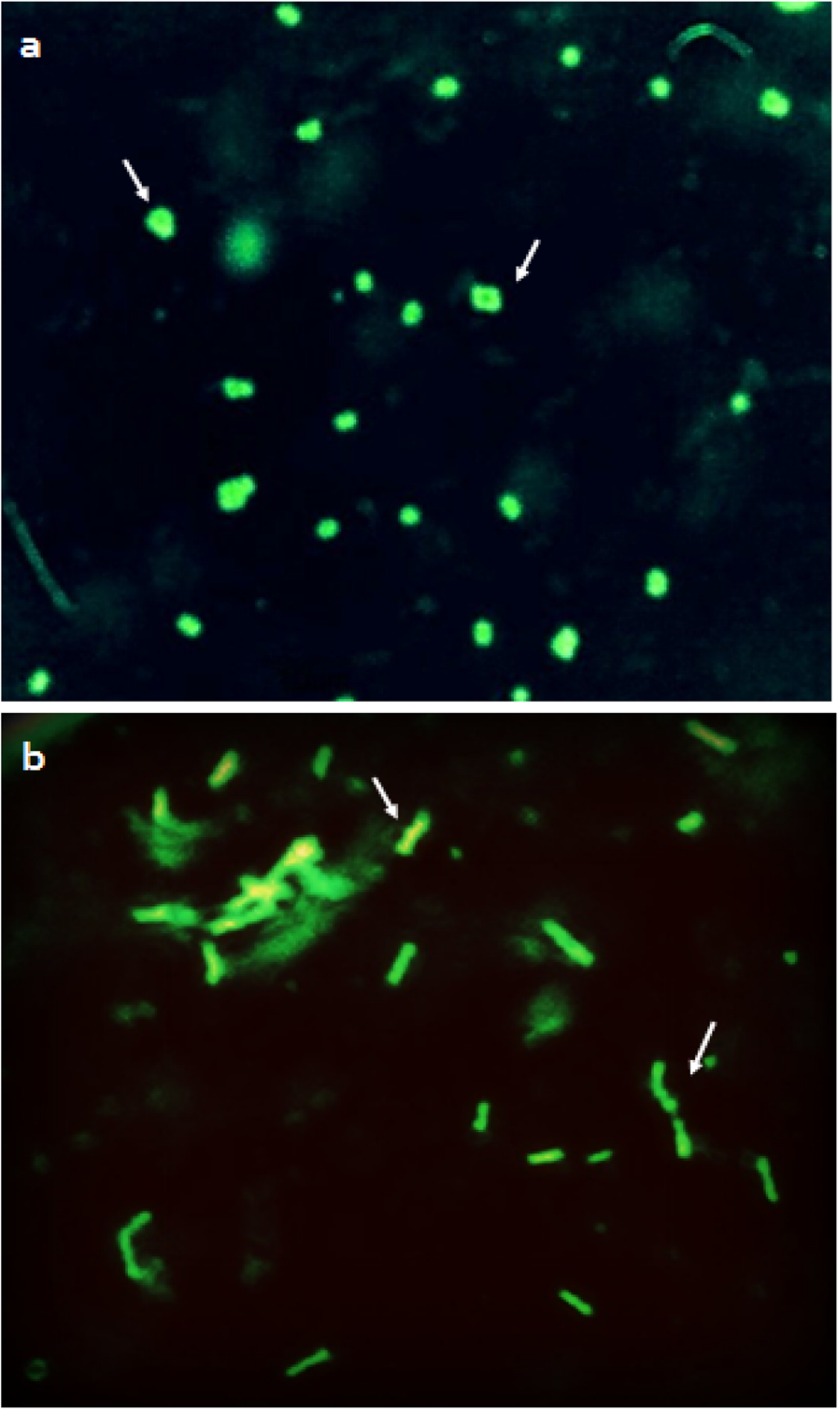
Cells of host LAB strains under fluorescence microscope (× 3000). **a** *P. acidilactici* MTCC 5101. **b** *L. brevis* MTCC 1750. White arrows depict tetrads clusters of *P*. *acidilactici* and rods of *L*. *brevis*

The transformation of LAB strains in this study with plasmid expressing *gfp* gene under constitutive promoter for erythromycin gene resulted in visible fluorescence in colonies under UV light. Figures 5 and 6 show the bacterial colonies under UV light and bacterial cells under a fluorescence microscope equipped with excitation and emission filters for GFP. Notably, the strains fluoresced only transiently, and no toxicity of GFP for the bacterial hosts and no protein degradation in cell extracts or structural instability of the plasmid were observed.

### Bile salt hydrolase activity

**Fig. 7:**
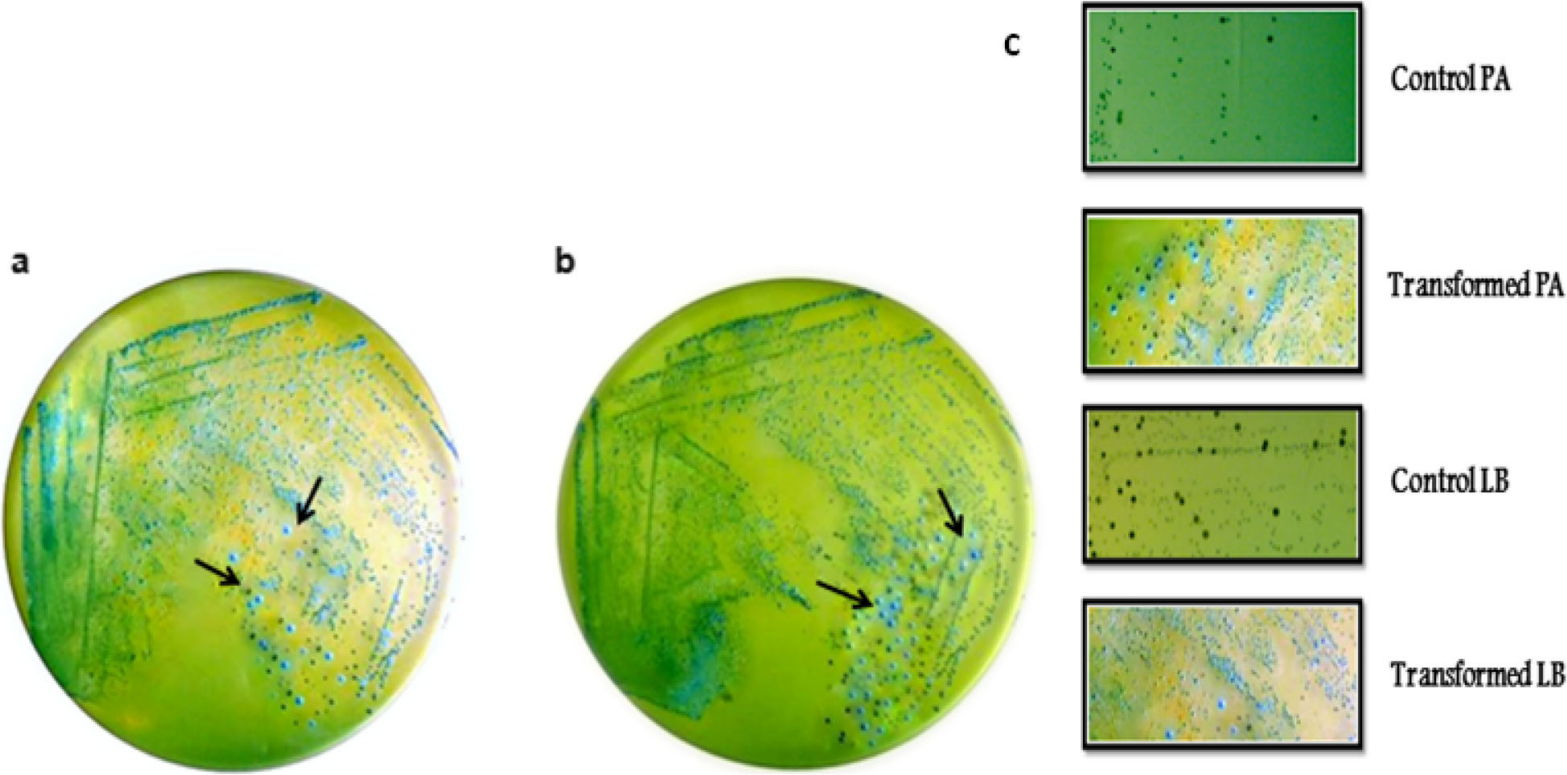
Bile salt hydrolase plate assay showing white precipitates around BSH^+^ colonies marked by arrows. **a** *P. acidilactici* MTCC 5101. **b** *L. brevis* MTCC 1750. **c** Bacterial colonies in control (0.3% bile concentration) and transformed LAB cultures (0.5% bile concentration)

In direct plate assay for BSH, the growth of transformed LAB strains on MRSA plates with 0.5% bile salt mixture resulted in white precipitates around active bacterial colonies due to precipitation of free bile acids (Fig. 7). The plasmid cured host strains served as a negative control marked with absence of inhibition zone and original isolate; *P. acidilactici* MTCC 5101 harboring native pCP289 plasmid with pediocin operon was used as a positive control. The results indicated fewer Bsh^+^ colonies in plates with *L. brevis* MTCC 1750 as compared to *P. acidilactici* MTCC 5101, possibly due to strain-specific expression of BSH.

BSH activities of the LAB strains transformed with pPBT-GFP were measured towards sodium glycocholate (GC), sodium taurocholate (TC), sodium taurodeoxycholate (TDC), and bile salt mixture using quantitative ninhydrin assay (Table 5). Values were found to be identical with a specific activity of BSH from *L. fermentum* NCDO 394 previously reported by Kumar et al. [36] against the listed bile salts. Since BSH enzymes extracted from different sources tend to have different substrate specificities [34, 37, 40], both the strains showed a high specific enzyme activity towards glycine-conjugated bile salts as compared to tauro-conjugated salts. *P. acidilactici* MTCC 5101 showed a higher BSH-specific activity towards all the three bile salts as compared to *L. brevis* MTCC 1750 as evident from ninhydrin assay.

**Table 5:** Specific bile salt hydrolase (BSH) activity of LAB strains

LAB strains transformed with pPBT-GFP	BSH activity^a^			
	Sodium glycocholate (GC)	Sodium taurocholate (TC)	Sodium taurodeoxycholate (TDC)	Bile salt mixture
	Specific activity (U/mg)	Specific activity (U/mg)	Specific activity (U/mg)	Specific activity (U/mg)
*P. acidilactici* MTCC 5101	126.1 ± 10.6	68.1 ± 7.6	90.2 ± 8.7	128.3 ± 9.7
*L. brevis* MTCC 1750	95.4 ± 4.2	43.4 ± 5.3	35.5 ± 9.8	88.1 ± 6.3

^a^Values are means of triplicates ± standard error of means

## Discussion

Keeping biosafety aspect in view, a shuttle expression vector was developed for *P. acidilactici* MTCC 5101 and *L. brevis* MTCC 1750. Both LAB strains have been isolated from human food sources and can be safely used at commercial scale. The designed system included pediocin operon and green fluorescent protein as selectable markers, replication origins for two LAB strains based on theta-replication, and heterologous bile salt hydrolase gene. Vector derivation was accomplished by recombining desired fragments to construct vector pPBT-GFP, followed by electroporation into plasmid cured LAB strains. Transformants with Ped^+^, GFP^+^, and BSH^+^ phenotype containing vector pPBT-GFP were screened on MRSA plates.

LAB plasmids are extremely diverse in terms of size (from 0.87 kb to more than 250 kb), copy number (from 1 to more than 100 plasmids per cell), and phenotypes (sugar fermentation, bacteriocin production, etc.) conferred on their hosts [12]. Large-sized plasmid usually have lower copy numbers, and in order to ensure proper distribution of plasmid copies to both daughter cells, a partition system has been evolved in the case of low copy plasmids [41]. Control of the plasmid copy number is achieved by modulating the intracellular concentration of the initiator protein with negative regulatory circuits that may include antisense RNA, both antisense RNA and proteins, and sites for binding initiator protein [42]. Plasmids with theta-type replicons tend to have a narrower host range than the rolling circle counterparts. However, a narrow host range is advantageous in minimizing the chances of horizontal transfer of plasmids among bacteria, thus, making them safer in terms of containment. These safety aspects of plasmid based vectors are very significant while handling LAB strains of commercial value [39]. In addition to the safety aspects of theta-replicating plasmids, these are also more stable both structurally and segregationally and can be used for cloning long DNA inserts [29, 42]. Shuttle vector pPBT-GFP was designed based on two theta-type replicons and was able to maintain itself for a fair number of generations (~ 50) in both the hosts. The copy number of pPBT-GFP in *P. acidilactici* MTCC 5101 and *L. brevis* MTCC 1750 was found to be 4.4 and 2.8, respectively, which revealed that pPBT-GFP has a low copy number and is stable in host cells. Therefore, the theta-type replication shuttle vector, pPBT-GFP, with its characteristics of narrow host range and stability in host cells may serve as an ideal tool for improving LAB strains of commercial value using genetic engineering.

Most laboratories incorporate antibiotic-resistant genes in vectors for ease of screening and selecting for transformants and maintain the genetic modification of interest. However, strains to be applied in the food industry cannot potentially carry transferable antibiotic resistance markers. To address this issue, the design and development of markers other than antibiotic resistance genes are a requisite for promoting industrial use of these bacteria. Selectable markers based on specific heterologous genes from other LAB, like resistance to heavy metals, production of antimicrobial bacteriocin-like substances, and utilization of certain complex carbohydrates, have been developed [43–49]. Table 6 lists the plasmid vectors used for cloning in *P*. *acidilactici* and *L. brevis* strains. Most of these plasmids encode antibiotic resistance genes as selectable marker which is a major safety concern for food-based applications. Therefore, in the present study, a shuttle vector pPBT-GFP was constructed without any antibiotic resistance marker genes. In contrast, genes encoding pediocin production and secretion have been incorporated, which has an effective wide-ranging antimicrobial activity, making it a promising and reliable system for industrial applications.

**Table 6:** Vectors used for cloning in strains of *P*. *acidilactici* and *L*. *brevis*

Plasmid	Host strain(s)	Size (kb)	Relevant characteristics	Reference
pRS4 derivatives (pRS4C1-C3)	*P. pentosaceus* *P. acidilactici* *L. plantarum* *L. casei* *E. coli*	7.8	Amp^R^, Cm^R^	[21]
pAMJ*AB*	*L. lactis* MG1363 *P. acidilactici, E. coli*	9.0	Em^R^ Pediocin PA-1 *pedA* Immunity gene *pedB*	[22]
pRS5	*P. pentosaceus* *E*. *coli*	10.1	Amp^R^, Cm^R^	[23]
pLES003	*L. brevis* *E. coli*	6.1	Amp^R^, Em^R^	[24]
pCP289	*P. acidilactici* MTCC 5101	9.2	Ped operon RepB ori	Present study
pPBT-GFP	*P. acidilactici* MTCC 5101 *L. brevis* MTCC 1750	9.6	Ped operon, RepA, ori LB, RepB ori PA, GFP, BSH	Present study

Apart from pediocin, green fluorescent protein (GFP) is a commonly employed selectable marker with potential ease of visualization as an advantageous trait when compared with other reporter proteins. The safety of GFP protein has been evaluated in in vivo studies in mice [50] and has also been used to tag LAB strains [32]. Although a majority of studies have reported a very strong fluorescence in the case of *E. coli* strains transformed with the *gfp*-carrying plasmids under the control of constitutive promoters, very few positive reports are available in the case of different LAB strains [51]. GFP was expressed in LAB strains *P. acidilactici* and *L. brevis*, using the vector pPBT-GFP under the control of a constitutive erythromycin (*ery*) gene promoter derived from vector pH 2515 [52]. A strong and consistent fluorescence due to GFP can only be observed in colonies synthesizing massive quantities of protein. On the other hand, a low-level expression can be studied using epifluorescence microscopy and western blotting. This phenotype thus seemed essentially correlated with the amount of GFP produced. A strong fluorescence was observed for both LAB strains transformed with pPBT-GFP. The promoter sequence does not encode any antibiotic resistance protein and was used for a stable and strong expression of *gfp* gene placed next to it. Notably, a transient fluorescence was observed with no toxicity of GFP observed to the bacterial hosts and no reduction in colony numbers as compared to non-transformed bacterial cells.

Probiotic LAB strains have been reported to possess hypocholesterolemic properties mainly attributable to the production of the enzyme and bile salt hydrolase (BSH). Most of the probiotic LAB strains show bile tolerance or resistance to varied levels and BSH enzymes have been purified and characterized from a number of LAB species [36, 53, 54]. These enzymes belong to choloylglycine hydrolase family of enzymes and have been classified as N-terminal nucleophilic (Ntn) hydrolases with an N-terminal cysteine residue, which serves as the nucleophile and proton donor in the catalytic process [55]. An autoproteolytic reaction leads to the removal of initiation formyl methionine, thus making Cys-1 residue the catalytic center in Ntn hydrolase superfamily [56] and removal or replacement of N-terminal Cys with other potential nucleophilic residues, Ser or Thr, generated an inactive enzyme [57–59]. The present study describes cloning and heterologous expression of intracellular BSH enzyme produced in *L. fermentum* NCDO 394 using shuttle vector pPBT-GFP. *P. acidilactici* and *L. brevis* are also acid- and bile-tolerant strains and may contain gene(s) for bile salt hydrolase as well [16, 33, 53, 60]. However, limited data is available as to the characterization of BSH in these strains; incorporation of heterologous *bsh* gene via cloning may also be considered as an enhancement strategy by introduction of an extra copy of *bsh* gene in addition to the existing one. Recombinant BSH was successfully produced and secreted using *L. lactis* Usp45 signal peptide under constitutive *ery* gene promoter, the same as the one used for GFP expression. Usp45 signal peptide is commonly used for heterologous protein secretion in *L. lactis*. The sec pathway is a ubiquitous secretion system which is based on the translocation of protein across the membrane, followed by cleavage of N-terminal signal peptide by a signal peptidase, thus releasing the protein into the medium [61]. The nucleotide sequence analysis of gene encoding Usp45 protein indicated the presence of a 1383 bp open reading frame encoding 461 amino acid long protein with a 27 amino acid signal peptide at N-terminal [62] which undergoes cleavage, releasing the mature protein extracellularly [63]. van Asseldonk et al. [64] reported a very high secretion efficiency for Usp45 signal peptide and has been used for the secretion of heterologous proteins in a number of Gram-positive LAB host strains since then [65, 66]. The successful secretion of BSH in the present work indicated that the Usp45 signal peptide is functional in *Pediococcus* and *Lactobacillus* species as well.

Since the presence of bile imposes stress towards the growth and survival in majority of LAB species, this mechanism can also be used as a selective pressure for plasmid maintenance in the host LAB strains [67, 68]. In the present study, *P*. *acidilactici* MTCC 5101 was observed to be sensitive to bile salts at 0.3% concentration in the medium; however, on transformation, the strain was found to maintain its growth even at 0.5%. Thus, 0.5% concentration can be used as a selective pressure for selection and maintenance of the vector in the transformants. This also obviates the need for use of any other selective pressure for the maintenance of the plasmid vector. Specific BSH activity revealed that substrate preference of BSH enzyme is more inclined towards glycine-conjugated bile substrates as compared to taurine-conjugated salts, consistent with the data already reported in literature for other LAB BSH enzymes [36, 37, 54, 59, 69–71]. BSH from *L. fermentum* NCDO 394 has already been well characterized by Kumar et al. [36] and found to be stable and functional in a range of pH and temperature values identical to the human body.

Hence, LAB hosts carrying shuttle vector pPBT-GFP with a stable copy number and narrow host range can be efficiently used as cholesterol-lowering probiotic strains for use in functional foods at an industrial scale too. Such genetic tools also can be employed for the production of various heterologous proteins in food-based systems for imparting traits with beneficial effects for humans*.* Since pediococci are usually associated with vegetables and meat products and are observed to grow poorly in dairy or milk products, the present study selected a dairy-based system in *L. brevis* to be used as shuttle host for applications involving dairy products.

The present study was designed for developing expression systems for pediococcal strains due to limited availability of vectors for genetic manipulation in species of the genus *Pediococcus* (Table 6). Studies have reported probiotic characteristics of various pediococcal strains isolated from varied habitats [20, 60, 72–74], which further emphasizes on the need for improvement in members of this genus. Strains of *L. brevis* also display a limited availability of cloning and expression systems for the production of recombinant products. Hence, the shuttle vector pPBT-GFP was designed to widen the industrial potential of such strains by genetic improvement. Such efficient and stable expression vectors are a must nowadays, and research must be geared towards the development of efficient vectors for LAB strains to be used in food based applications.

## Conclusion

This research work reports the development of a LAB/LAB shuttle vector pPBT-GFP efficiently replicating in LAB species, *Pediococcus acidilactici* MTCC 5101 and *Lactobacillus brevis* MTCC 1750. Genes for the production and expression of pediocin and green fluorescent protein were used as selection markers. Heterologous gene-encoding bile salt hydrolase with cholesterol-lowering potential was successfully cloned and secreted using Usp45 signal peptide mediated secretory pathway.

## Supplementary information

**Additional file 1:** **Figure S1.** Agarose gel electrophoresis of various plasmids used in the study, (a) Lane 1: NEB Supercoiled DNA ladder; Lane 2: Shuttle vector pPBT-GFP; Lane 3: Plasmid pLES003; Lane 4: Plasmid pCP289; Lane 5: Vector pMK-RQ. Multiple bands are visible due to supercoiled, partially nicked and linear forms of plasmids. **Figure S2.** Agarose gel electrophoresis of DNA fragments used for vector development, Lane 1: Novagen Perfect DNA ladder; Lane 2: Pediocin operon; Lane 3: oriLB; Lane 4: oriPA; Lane 5: bsh; Lane 6: gfp. Sequence of Shuttle Vector pPBT-GFP, 9.6 kb.

## Data Availability

All data generated or analyzed during this study are included in this published article and its supplementary information files**.**

## Abbreviations

BSA: Bovine serum albumin
BSH: Bile salt hydrolase
GFP: Green fluorescent protein
GRAS: Generally recognized as safe
LAB: Lactic acid bacteria
MTCC: Microbial Type Culture Collection
NCBI: National Centre for Biotechnology Information
UV: Ultraviolet
w/v: Weight/volume
