# Correction to: Construction of a shuttle expression vector for lactic acid bacteria

**DOI:** 10.1186/s43141-020-00056-4

**Published:** 2020-08-04

**Authors:** Tejinder Kaur, Praveen P. Balgir, Baljinder Kaur

**Affiliations:** Department of Biotechnology, Punjabi University, Patiala, 147002 India

**Correction to: J Genet Eng Biotechnol 17, 10 (2019)**

**https://doi.org/10.1186/s43141-019-0013-4**

Following publication of the original article [1], the authors reported that there is an error in the Acknowledgements section. The correct Acknowledgements section is:

**Acknowledgements**

The authors would like to acknowledge DST and ICMR, New Delhi, for the financial assistance to PPB and TK. The authors are grateful to Prof. Masanori Sugiyama and Masafumi Noda of Hiroshima University, Japan for providing plasmid pLES003.

This has now been included in this correction article.

## References

[1] Kaur T, Balgir PP, Kaur B (2019). Construction of a shuttle expression vector for lactic acid bacteria. J Genet Eng Biotechnol.

