# Supplementary material for: Construction of a shuttle expression vector for lactic acid bacteria
Source: J Genet Eng Biotechnol. 2019 Nov 18;17:10. doi: 10.1186/s43141-019-0013-4 (PMC6859148; doi:10.1186/s43141-019-0013-4)
Supplement: Supplementary file 1 — Additional file 1: Figure S1. Agarose gel electrophoresis of various plasmids used in the study, (a) Lane 1: NEB Supercoiled DNA ladder; Lane 2: Shuttle vector pPBT-GFP; Lane 3: Plasmid pLES003; Lane 4: Plasmid pCP289; Lane 5: Vector pMK-RQ. Multiple bands are visible due to supercoiled, partially nicked and linear forms of plasmids. Figure S2. Agarose gel electrophoresis of DNA fragments used for vector development, Lane 1: Novagen Perfect DNA ladder; Lane 2: Pediocin operon; Lane 3: oriLB; Lane 4: oriPA; Lane 5: bsh; Lane 6: gfp. Sequence of Shuttle Vector pPBT-GFP, 9.6 kb. [file 43141_2019_13_MOESM1_ESM.doc]

**
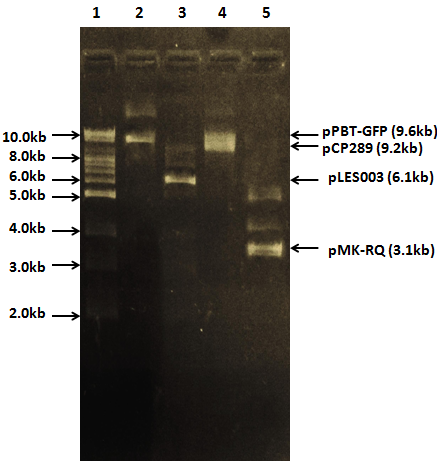
**

**Additional file 1: Figure S1 Agarose gel electrophoresis of various plasmids used in the study, (a) Lane 1: NEB Supercoiled DNA ladder; Lane 2: Shuttle vector pPBT-GFP; Lane 3: Plasmid pLES003; Lane 4: Plasmid pCP289; Lane 5: Vector pMK-RQ. Multiple bands are visible due to supercoiled, partially nicked and linear forms of plasmids**

**
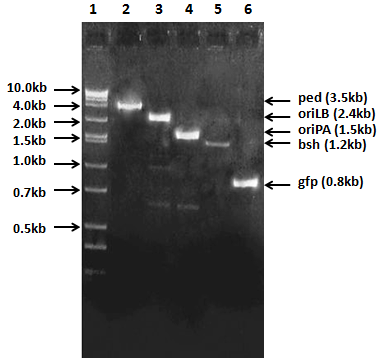
**

**Figure S2 Agarose gel electrophoresis of DNA fragments used for vector development, Lane 1: Novagen Perfect DNA ladder; Lane 2: Pediocin operon; Lane 3: oriLB; Lane 4: oriPA; Lane 5: bsh; Lane 6: gfp**

**Sequence of Shuttle Vector pPBT-GFP, 9.6kb**

GGATCCTATCTAACTAATACTTGACATTTAAATTGAGTGGGAACTAGAATAAGCGCGTATTAAGGATAATT

*pedA*

TAAGAAGAAGGAGATTTTTGTG**ATGAAAAAAATTGAAAAATTAACTGAAAAAGAAATGGCCAATATCATTGGTGGTAAATACTACGGTAATGGGGTTACTTGTGGCAAACATTCCTGCTCTGTTGACTGGGGTAAGGCTACCACTTGCATAATCAATAATGGAGCTATGGCATGGGCTACTGGTGGACATCAAGGTAATCATAAATGCTAG**CA

*pedB*

TTATGCTGAGCTGGCATCAATAAAGGGGTGATTTT**ATGAATAAGACTAAGTCGGAACATATTAAACAACAAGCTTTGGACTTATTTACTAGGCTACAGTTTTTACTACAGAAGCACGATACTATCGAACCTTACCAGTACGTTTTAGATATTCTGGAGACTGGTATCAGTAAAACTAAACATAACCAGCAAACGCCTGAACGACAAGCTCGTGTAGTCTACAACAAGATTGCCAGCCAAGCGTTAGTAGATAAGTTACATTTTACTGCCGAAGAAAACAAAGTTCTAGCAGCCATCAATGAATTGGCGCATTCTCAAAAAGGGTGGGGCGAGTTTAACATGCTAGATACTACCAA**

*pedC*

**TACGTGGCCTAGCCAATAG**TACTGATAAAGGGGATATTGTAG**TTGTCTAAGAAATTTTGGTCAAATATCTTTTTAGCATTAGGCGTCTTTCTTGCTTTTGCAGGAGTTGCTACCATATCGGTGAGTGCTGACAGTTCCGCTACTATAGAATCAAATACTAGCTCGAAAATCATCGATGGTGCAACTTATGAAGAAAACATCAGGGGCGTTATTCCTATTACGCTAACTCAATATTTGCATAAAGCTCAAACTGGAGAAAAATTTATTGTCTTTGTCGGGTTCAAGGAGTGTGTGCATTGTCGTAAATTTTCTCCAGTCATGAAACAGTACTTACAACAAAGTCAGCATCCCATTTATTACTTAGACTATGGGAACAACGGGTCTTTCAGCATGGCTTCTCAAAAACAAATAACTGATTTCTATTCAACTTTTGCAACCCCCATGAGTTTTATGGGAACGCCAACTGTTGCCTTGCTCGATAATGGTAAGGTGGTATCAATGACCGCTGGTGATGATACCACTTTATCTGATTTACAACAGATTACTGCTGATTACAATAATCAGTAG**TCACCTGGTTAATATGGTTTTGTAACCAATGTAAAAGGCGATGGATCTTTGAAATCGTCTTTTTTTATGCAC

*pedD*

AAATTTTAAAGATCGGTGGTTTGCTT**ATGTGGACTCAAAAATGGCACAAATATTATACAGCACAAGTTGATGAAAATGACTGTGGTTTAGCTGCACTAAATATGATCCTAAAATACTATGGCTCCGATTACATGTTGGCCCATCTTCGACAGCTTGCCAAAACAACTGCTGACGGTACAACTGTTTTGGGGCTTGTTAAAGCAGCAAAACACTTAAATTTAAATGCCGAAGCTGTGCGTGCTGATATGGATGCTTTGACAGCCTCACAATTGCCATTACCAGTCATTGTTCATGTATTCAAGAAAAATAAGTTACCACACTACTATGTTGTCTATCAGGTAACTGAAAACGATTTAATTATTGGTGATCCTGATCCAACCGTTAAAACCACTAAAATATCGAAATCACAATTTGCTAAAGAATGGACCCAGATTGCAATTATCATAGCCCCAACAGTTAAATATAAACCCATAAAAGAATCACGGCACACATTAATTGATCTAGTGCCTTTATTGATTAAACAAAAAAGATTAATTGGACTAATTATTACCGCAGCAGCTATAACAACATTAATCAGTATTGCTGGTGCATATTTCTTTCAGTTAATTATCGATACTTATTTGCCGCACTTGATGACTAATAGGCTTTCACTAGTTGCCATTGGTCTGATTGTAGCTTATGCTTTCCAAGCAATTATCAACTATATACAAAGTTTTTTTACGATTGTATTAGGACAACGTCTCATGATCGACATCGTTTTAAAATACGTTCACCATCTTTTTGATTTACCAATGAATTTTTTTACTACCCGTCATGTCGGTGAAATGACCTCACGCTTTTCTGATGCAAGCAAAATTATTGATGCACTTGGAAGTACAACGCTCACCCTTTTTTTAGACATGTGGATTTTATTAGCAGTAGGGTTATTTTTGGCCTATCAAAACATCAATTTATTTTTATGCTCGTTAGTTGTGGTTCCAATTTACATCTCGATTGTTTGGCTATTTAAAAAAACTTTTAATCGTTTAAATCAAGATACAATGGAAAGCAATGCAGTTCTTAATTCTGCTATTATTGAAAGTCTCAGTGGCATAGAAACCATTAAATCACTAACTGGTGAAGCAACTACAAAAAAAAAGATTGACACACTATTTTCTGACTTATTGCATAAAAACTTGGCTTATCAAAAAGCTGATCAAGGACAACAAGCTATCAAAGCAGCTACTAAATTAATCCTAACTATTGTTATCCTTTGGTGGGGTACTTTTTTTGTTATGCGACACCAACTGTCTTTAGGTCAGCTGTTAACTTATAATGCTTTGCTCGCTTACTTCTTGACCCCATTAGAAAATATTATTAATTTACAGCCTAAACTACAAGCTGCCAGAGTGGCTAATAATCGATTAAATGAGGTTTATCTAGTAGAGTCTGAATTTTCTAAATCTAGGGAAATAACTGCTCTAGAGCAACTAAATGGTGATATTGAGGTTAATCATGTTAGTTTTAACTATGGCTATTGTTCTAATATACTTGAGGATGTTTCTCTAACAATTCCACATCATCAGAAGATTACTATTGTAGGCATGAGTGGTTCGGGGAAAACGACCCTAGCCAAGTTGCTAGTTGGTTTTTTTGAGCCTCAAGAACAGCACGGTGAAATTCAGATTAATCATCACAATATATCTGATATTAGTCGCACAATTTTACGCCAATATATTAATTATGTTCCTCAAGAACCTTTCATTTTTTCGGGCTCTGTATTAGAAAATTTATTGTTAGGTAGCCGTCCTGGAGTAACTCAACAAATGATTGATCAAGCTTGTTCCTTTGCTGAAATCAAAACTGATATAGAAAATTTGCCTCAAGGTTATCATACTAGATTAAGTGAAAGTGGATTCAACTTATCTGGTGGGCAAAAACAGCGGTTATCAATAGCTAGAGCATTATTGTCTCCGGCACAATGTTTCATTTTTGACGAATCAACCAGT**

**AATTTAGACACCATTACTGAACATAAAATAGTCTCTAAGCTATTATTCATGAAAGACAAAACGATAATTTTTGTAGCACATCGTCTCAATATTGCGTCTCAAACCGATAAAGTTGTCGTTCTTGATCATGGAAAGATTGTTGAACAGGGATCACATCGACAATTGTTAAATTATAATGGGTATTATGCACGGTTAATTCATAATCAAGAATAG**CCTGACAAGAACCAGTCTGCTATTGATAGACTATTCTTGTCCGTGAAATCCTCGCGGCATGCCGATTTCGCTTCGCAAAACGGCACAAATTCAAGCCCGAAAACCTGTCCTTTTTTTGCAAAAAAAGGACACAAAAAAAACCGCACAAAAAAAGCCGAAAGTTTGCTGTTCGCAAATCGGCTTTTTAAGGCATTATGATTGCTCATTTTCTGTTTGAAACTGACTCAACCATTGGCTAACATCTTTCAAGTCTTCGATTTTCCAGAAGTTGCCGTTATAAGTCATAGCATTAGCAATGTTTTGTAGTTGCTGAAATTGTTCTGTAGTTAAAGTTGGTGAAGCCGTTTCTCTTTTTTCAAAAACTGGCGTATGGTCGAGCATATTTTCGGCTGTAGTTATATTTTTAGCGCCAGTTTTTTTGTAAAATTTACGGCCAAGCTTCTCATATTCCTTCGCTAGTGCCTTGCGCTTACGCACTTTCAGTTTCTTTTCTAGTT

*repA*

TCGCAATACGTTCTAATTCGG**TCATCGTAATCCTCCGTTTCGTAGTTTTTCGAGTATTTTTTGTCGCTCCTCTGCACGAATCTGTTCTTCGCGCGCCTCTTGTTCTTGTTTATTATGTTCCGCTTCGGTCGTTCCTAGCTTGAGACCTTTGATTTTATCAATTGCCCGCCATTTTTCTTTGTCGGACAGTTCGCCATTGTGCTCGATATTGAATTTGGCTTTCTTCAGGCGTTCAGTCTTGCTGACGTGGACATCTTCGGCATCTTTGCGTTCAGGTTTCCATGCAAAAGAATAGCCAATAACAGGCTTTCCTCGCCCTTTACCGTATTTCTTGCGGACAGTTAGCCCACGAAATAGGGGGGTAAGTTCTTCTTTGATTGGACTAATAACATATTGGTCGGTGTTAGACTGCCGGTAACTTTTTGGAATATCAAGCAATTCAATAAAATCGGATTTTGAAAAATAGGCGTAACCGGTGGTGCGATAACCTTTCAACAAACGGAACATCGTTTTAGCATAGCTGCTCTTCAAGTCACGAAACTCGACTAGGGCGTATCGTACCCAGCTCTCCAGATGGTTTAGCAAGGGAAGTGCATCTTTGTAAATCTCTATGTCAACATATGGTTCTTCCACATCACCGTTGATTTTAAATTTGGTAAACATGACAAATCTTTCAAATGACAACCCGCTTTTACTTTGGCTACCGAAACGCAAGTCCATCAAATGATTGTAAGTGCGCTTGAGGTCGTCAATAAATCGGCGATTGGCGGTCGGTTTGTAGTTACTAAGGTCTTTCAGCTGGTCAAAGCTAAAGCGTACAGTCTGGTCGCCCTTATCCCGCATACGACTAATGATCGAAAAGAACAGATTCATTTCTGTAGGACTGAATTTACGGAGTGGAATGGTGTTCAGTTCGGGTTGATATTTCACTAACTCATTGCTCAT**ATAATCACCTCAAAATTATTATAGCATATATAAAAGGATTTCATGCCCAAATTAACATGCCTTTAAACAGACAATTCCATGCCTTTAAACAGACAATTCCATGCCTTTAAACAGACAATTCCATGCCTTTAAACAGACAATTCCATGCCTTAATCATCCTGTACCCCTTGGGAGAGTAAGGTAGAACACCCCCTTAAAAGAGGTTTTAAAAGAGGTTTTAAAAGAGGTTATATAAGAGGCGGGGATCTCCGACTTCGTCTCCGTGCCCCAAAATGCCAAATCAGGACCAAAAACCAAAAAGGAGTGAGCGAAAATCACGCTCACATTTTGTTTGCAGCGTTCACTTCGTTCACTCGCTAAATTCAAAATCAAAAGCGTGGTTCAGGAAAAGCCTCGACTGACGCTCGGCAGGTAAGCCCAGATAGGCATAAAAGGCCATACTTTGCGTTCTAAGCTCGTTTGGCGACTTCTTCGTCGTGCTCTTTTTGGTTTTAGGCAAGCAGTTCATTTGCTAGACAATTAGGTATCGCTACGCTCCCTAATTGTCTGGCAAATGGCTTGTAAGAAGGGCGCTACGCTCCCCTCTTAACTCCCCACAAAAATAGCCGATTACCTATTTGGCAACATCTATGAAAAGCGATCGATGTTGCCTTGTCAATCAACTAGATGTGACAATCGGTGCCCCTTCTTCACCAAAAATTTTGGTACGTTAATTTGCAAATTTAATTAATTAGATATAGAATTTAATCGAACATATGTTCGTATAATTATGTAGCGACAGACGGACGTCTAAGAAACCATTATTATCATGACATTAACCTATAAAAATAGGCGTATCACGAGGCCCTTTCGTCTCGCGCGTTTCGGTGATGACGGTGAAAACCTCTGACACATGCAGCTCCCGGAGACGGTCACAGCTTGTCTGTAAGCGGATGCCGGGAGCAGACAAGCCCGTCAGGGCGCGTCAGCGGGTGTTGGCGGGTGTCGGGGCTGGCTTAACTATGCGGCATCAGAGCAGATTGTACTGAGAGTGCACCATATGTAGAAGCAAACTTAAGAGTGTGTTGATAGTGCAGTATCTTAAAATTTTGTATAATAGGAATTGAAGTTAAATTAGATGCTAAAAATTTGTAATTAAGAAGGAGTGATTACAGATCTGATATCCCATGGGCGGCCGCCTCGAGAAGGAGTGATTAATGAAAAAGAAGATTATCTCAGCTATTTTAATGTCTACAGTGATACTTTCTGCTGCAGCC

*bsh*

CCGTTGTCAGGTGTTTACGCTGAATTC**ATGTGCACGAGCATCAACGTCATTGCCCAGGACGGCTACCACGTCTTGGGGCGGACAATGGACTGGGACGACCTCTTAGTGTCGCCCATCTTCACCCCCCGTCACTACCAGTTGGCCTCGGTCTTTGACCACCGGGTCCACGAAAATCCCTACGCCATCATCGGCGGTGGCTCGATCACCGAGCGGCGAACCGACGTTTCCGACGGGGTCAACGAGTTTGGCTTGATGGCCCAAAAGCTGACCTTTAAAAACGGCGCCCGCCTGGTTGACGAGCGTCACCCCGACAAGGTCCAACTGGCCGCCTTTGAGCTAATCTTTTACTTGCTAGGCCACTTTAAATCGGTGGCTGACGTCGCGGCGCACCTGGATCAAATCGAACTAATGAACGACGTCAACGCCGACGTGCCCTTTGGCTACTCCGAGCAACACTTTGTCTTGTCCGACCCCACCGGGCGTTGCGTGGTCATTGAACCCAGCGAGCACCCGCTCAAGCTGATCGATAACCCGCTCGGGATCATGACCAACATGCCCAAGTTCGACCACCAGCTGGAACGCCTGCAGGACTACCTGGACTTCACCCCGGACTTTTTGAACGGCACCCTGGCGCCGAACACCTTTCACGTCACCACCGGCAAGCTCTCGGGTAAGAAGACACCACCCGGTGCCTACACCCCCAAGGGGCG**

**CTACGTCCGGGCCGCCTACATGAAGGAACTGGCCGACCAACCGGCATCAAAAGACGAGGCGCTGGCCACCACCTGGCACCTGCTCGATTCGGTCACCGTCCCCAAGAGCAAGGCCCACCGGCCAACCTTCTCGGTGTACCGGGCGGCCACCGTTGCTGAGGACCGCACCTACTACTTCCAGTCCTACCACCAGGCCCAGGTGACCTCGGTAAAGCTGACTGACGACTTGTTAAAGCGAGCCACCCCGCTCGTCTTTGACACCGCCGACGTTTGGGCACCGGTGAAATTGAACTAA**GGAGCTCGCCCTCGTACAGTAGTGCATCTTTTAAACCTCTTTTATAAACCTCTTTTAAACCTCTTTTAAGGGCATGTTCCACGTTACTCTACCAAGCGTTTCACAAATGTTTGGGGGTCCAATTGTCTGTTTATGGGGGTCCAATTGTCTGTTTATGGGGGTCCCGTCCCGTCTTGAGGTTCCGTAGGGGTCCAATTGTCTG

*repB*

TTTATGGGGGTCCAATTGTCTGTTTAGTATGGGGGTCCCGTCTTGAGGTTCCGGCCTTGAT**ATGGTAGCCTCTGATTTAGGAGGTGGTTTTTTGAGCAACGAACTAGTTAAATATGATCCAGAGTTAAATACCATTCCACTTCGAAAATTTACTCCAGTTGAAATGAATTTATTTTTTTCGATTATTTCTCGTATGCGTGATAAGGGAGATCAGACCGTTCGTTTTACTTTTGATCAATTAAAAGAGTTAAGTGCTTATAAACCTACTGTATGGGGGTCCCGTCTTGAGGTTCCGGCCTTGATATGGTAGCCTCTGATTTAGGAGGTGGTTTTTTGAGCAACGAACTAGTTAAATATGATCCAGAGTTAAATACCATTCCACTTCGAAAATTTACTCCAGTTGAAATGAATTTATTTTTTTCGATTATTTCTCGTATGCGTGATAAGGGAGATCAGACCGTTCGTTTTACTTTTGATCAATTAAAAGAGTTAAGTGCTTATAAACCTACTGCAAATAACCGTTTTGAAGATGACATTCAGAGAACTTATGAAAAAATGATGGGATTACATTTTGGTAGACGAAGTAAAAGTGGCTTAAATCGAGAATTTTTTGTTATGTTTACCGAATTTGAAATTAAAGGCGAAGCTGAAATACCTTACGTTGATATCCGAGTTTATCCTAAAGCCTTACACTTACTAAACGATTTAGAAAGTTGGGTTCGTTATGCGTTGGCAGAGTTTAGAGATTTAAAAAGTAGTTACGCAAAAACAATGTTTCGGTTACTAAAACAATTTAGAACTACTGGGTACGCTTACTTTTCCAAAGCAGATTTTGATGAGTTACTTGATATTCCAAAAACTTATCGGCAAGGCGACATTAACAAAAAAGTGATAAAACCAATCAAAGAAGAACTTACCCCCCTATTTCGTGGGCTAACTGTCCGAAAGAAATACGGTAAAGGGCGAGGAAAGCCTGTTATTGGCTATTCGTTTACCTGGAAACCCGAAAAGAAAGACGCTAACGACTTCTCACAAGGTCAATTTCAAGATGAACGTCAAAAACTCTTTAATATTCAGCATAATGGCGAATTAACAGAACAGGAAAAATGGCGTGCCATTGATAAAGTTAAGGGGTTAACTTTAGGCTCTACTGAAAAGCAAGCATTGGCTGTCAAACAAGCCGAACATGATAAAAAAATAAGAGATCAAGCAAGAAAAGAAGCACTTGCTGAACTCCGAAAGGGGTTTGGAAATCATGCCTAA**AACAATTAGAGAACTTGCTGACGAATTGAAGGTCTCTAAACAAACTATTCAATACCACTACCAAAGACTACCAACAAAGAACCGACAGGTACCGGTACCAGTGTGTTGATAGTGCAGTATCTTAAAATTTTGTATAATAGGAATTGAAGTTAAATTAGATGCTAAAAATTTGTAATTAAGAAGGAGTGATTACAGATCTGATATCCCATGGGCGGCCGCCTCGAGAAG

*gfp*

GAGTGATTA**ATGGCTAAAGGTGAAGAATTATTTACTGGTGTTGTTCCAATTTTAGTTGAATTAGATGGTGATGTTAATGGTCAAAAATTTAGTGTTAGTGGTGAAGGTGAAGGTGATGCTACTTATGGTAAATTAACTTTAAAATTTATTTGTACTACTGGTAAATTACCAGTTCCATGGCCAACTTTAGTTACTACTTTTAGTTATGGTGTTCAATGTTTTAGTCGTTATCCAGATCATATGAAACAACATGATTTTTTTAAAAGTGCTATGCCAGAAGGTTATGTTCAAGAACGTACTATTTTTTATAAAGATGATGGTAATTATAAAACTCGTGCTGAAGTTAAATTTGAAGGTGATACTTTAGTTAATCGTATTGAATTAAAAGGTATTGATTTTAAAGAAGATGGTAATATTTTAGGTCATAAAATGGAATATAATTATAATAGTCATAATGTTTATATTATGGCTGATAAACCAAAAAATGGTATTAAAGTTAATTTTAAAATTCGTCATAATATTAAAGATGGTAGTGTTCAATTAGCTGATCATTATCAACAAAATACTCCAATTGGTGATGGTCCAGTTTTATTACCAGATAATCATTATTTAAGTACTCAAAGTGCTTTAGCTAAAGATCCAAATGAAAAACGTGATCATATGATTTTATTAGAATTTGTTACTGCTGCTGGTATTACTCATGGTATGGATGAATTATATAAATAA**
